# Acquired Hepatocerebral Degeneration After a Splenorenal Shunt in the Sub-Saharan Africa Context: A Case Report and Brief Review of Literature

**DOI:** 10.7759/cureus.23064

**Published:** 2022-03-11

**Authors:** Abdu Mohammed, Abate Bane, Getahun Mengistu, Fekadu Ayalew, Amir Sultan Seid

**Affiliations:** 1 Department of Internal Medicine, Division of Gastroenterology and Hepatology, Adera Medical Center, Addis Ababa, ETH; 2 Department of Internal Medicine, Division of Gastroenterology and Hepatology, Addis Ababa University, Addis Ababa, ETH; 3 Department of Neurology, Addis Ababa University, Addis Ababa, ETH; 4 Department of Radiology, Adera Medical Center, Addis Ababa, ETH

**Keywords:** liver cirrhosis, ethiopia, sub saharan africa, splenorenal shunt, non cirrhotic portal hypertension, acquired hepatocerebral degeneration

## Abstract

Acquired hepatocerebral degeneration (AHD) is a neurologic syndrome caused by liver dysfunction and long-standing portosystemic shunting. The pathogenesis of the condition is predominantly considered to be related to the deposition of manganese in parts of the brain due to shunting. We report a case of a 25-year-old male who underwent splenectomy and splenorenal shunt for recurrent upper GI bleeding (UGIB) due to esophageal varices caused by non-cirrhotic portal hypertension (NCPH). He presented with bradykinesia, hypophonia, gait instability, and rigidity of the lower extremities 18 months after the procedure was done.

## Introduction

Acquired hepatocerebral degeneration (AHD) is an unusual condition characterized by extrapyramidal symptoms, movement disorder, and cognitive manifestations in patients with chronic liver disease, especially those who develop portosystemic shunting spontaneously or induced surgically [1]. Patients typically present with dysarthria, ataxia, tremor, involuntary movements, and altered mental status and often do not respond to conventional medical therapy for hepatic encephalopathy. It is often missed as a possible cause of cognitive dysfunction in patients with liver disease. Its etiology has been posited to be related to the accumulation of metals in the brain, specifically manganese. T1-weighted MRI typically shows bilateral pallidal hyperintensities, likely related to the accumulation [2]. We report a case of a patient with likely features of AHD who presented with a movement disorder resulting from a shunt procedure for variceal bleeding.

## Case presentation

We present a case of a 25-year-old male diagnosed with non-cirrhotic portal hypertension (NCPH) with multiple large esophageal varices. He had recurrent episodes of upper GI bleeding (UGIB), requiring repeated sessions of variceal band ligation, and was put on non-selective beta-blockers for prophylaxis. He also had hypersplenism evidenced by pancytopenia and huge splenomegaly. For the overall condition, he underwent splenectomy and splenorenal shunt in India. His post-op course was complicated by splenic vein thrombosis, which required anticoagulation with warfarin for six months. However, after the shunt procedure, the patient had remarkable clinical improvement and was doing well.

**Figure 1:**
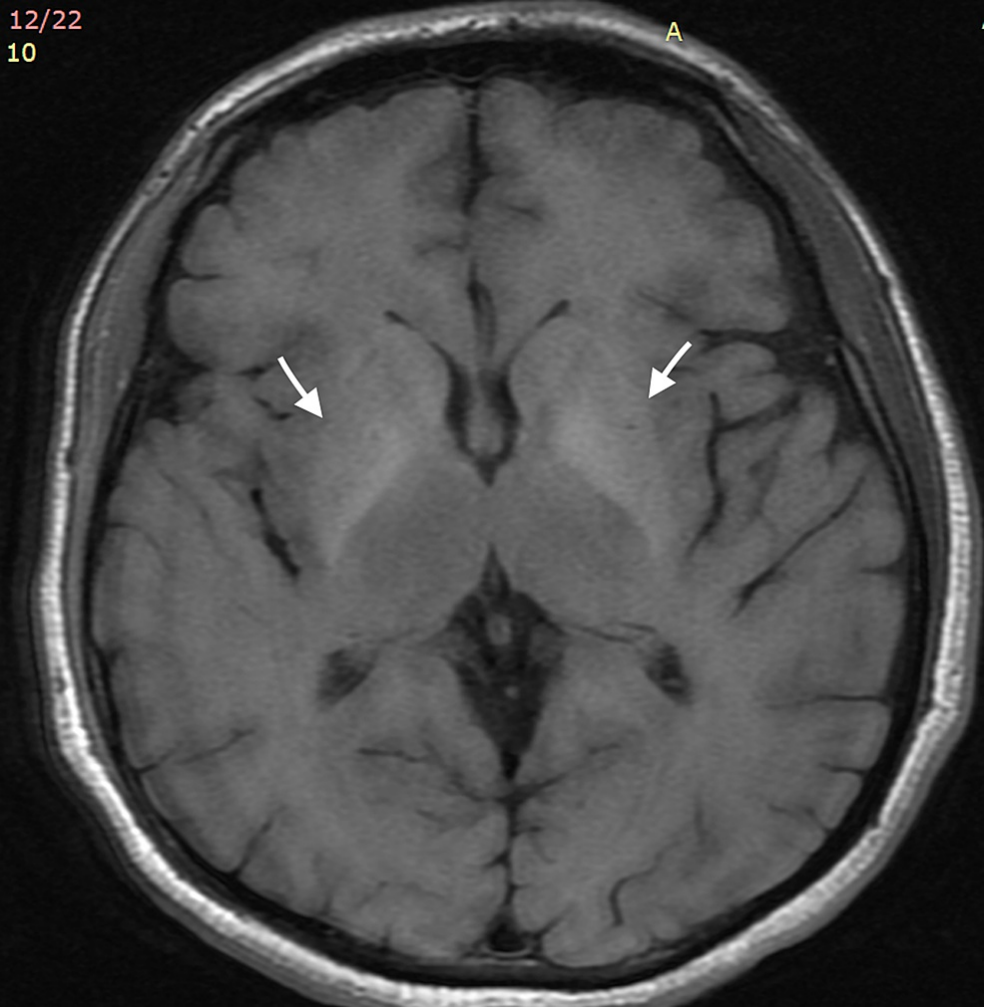
Axial T1-weighted MRI of the brain. MRI of the brain showing symmetrical hyperintensity on T1-weighted images in the globus pallidus (arrows).

**Figure 2:**
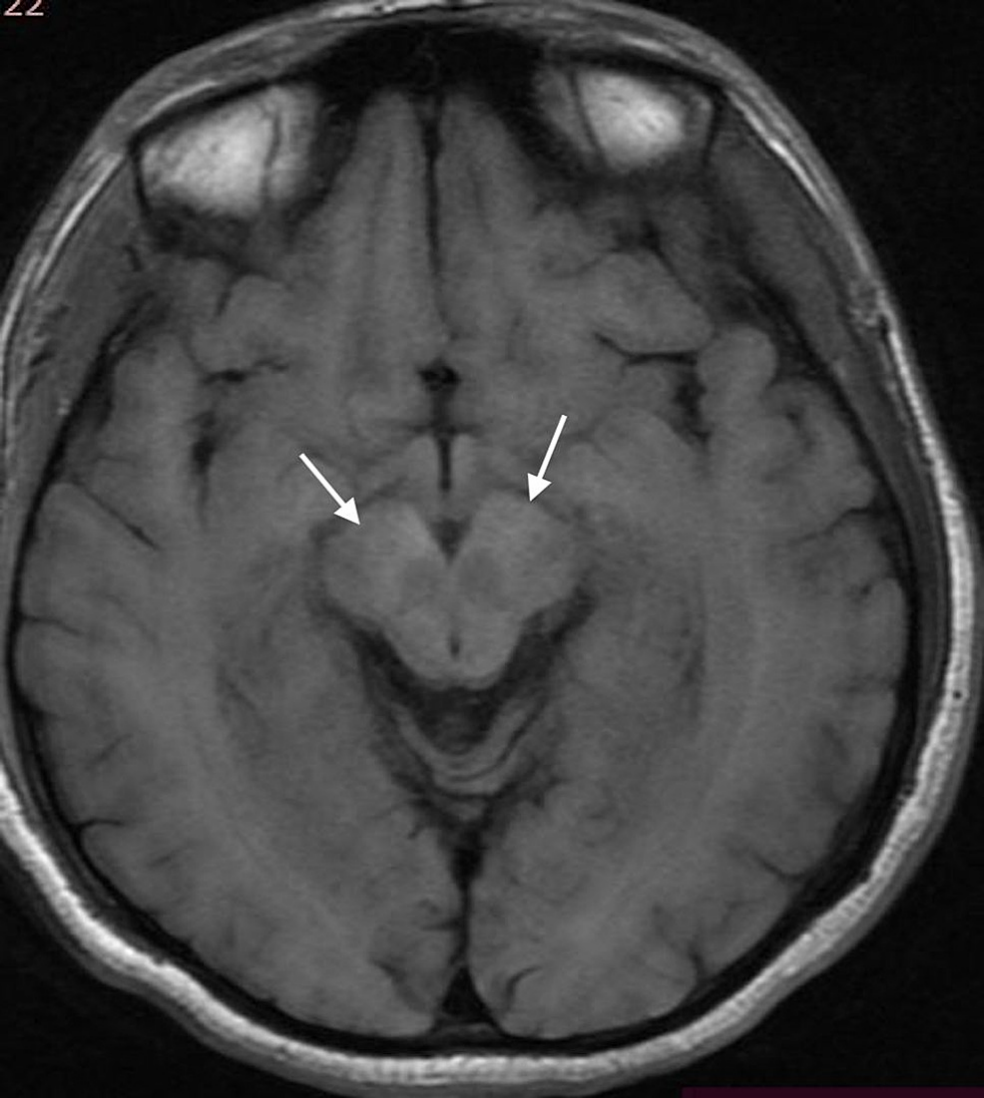
Axial FLAIR MRI image of the brain. MRI FLAIR image of the brain at the level of midbrain showing bilateral hyperintensity on the crura (arrows). FLAIR: Fluid-attenuated inversion recovery.

**Figure 3:**
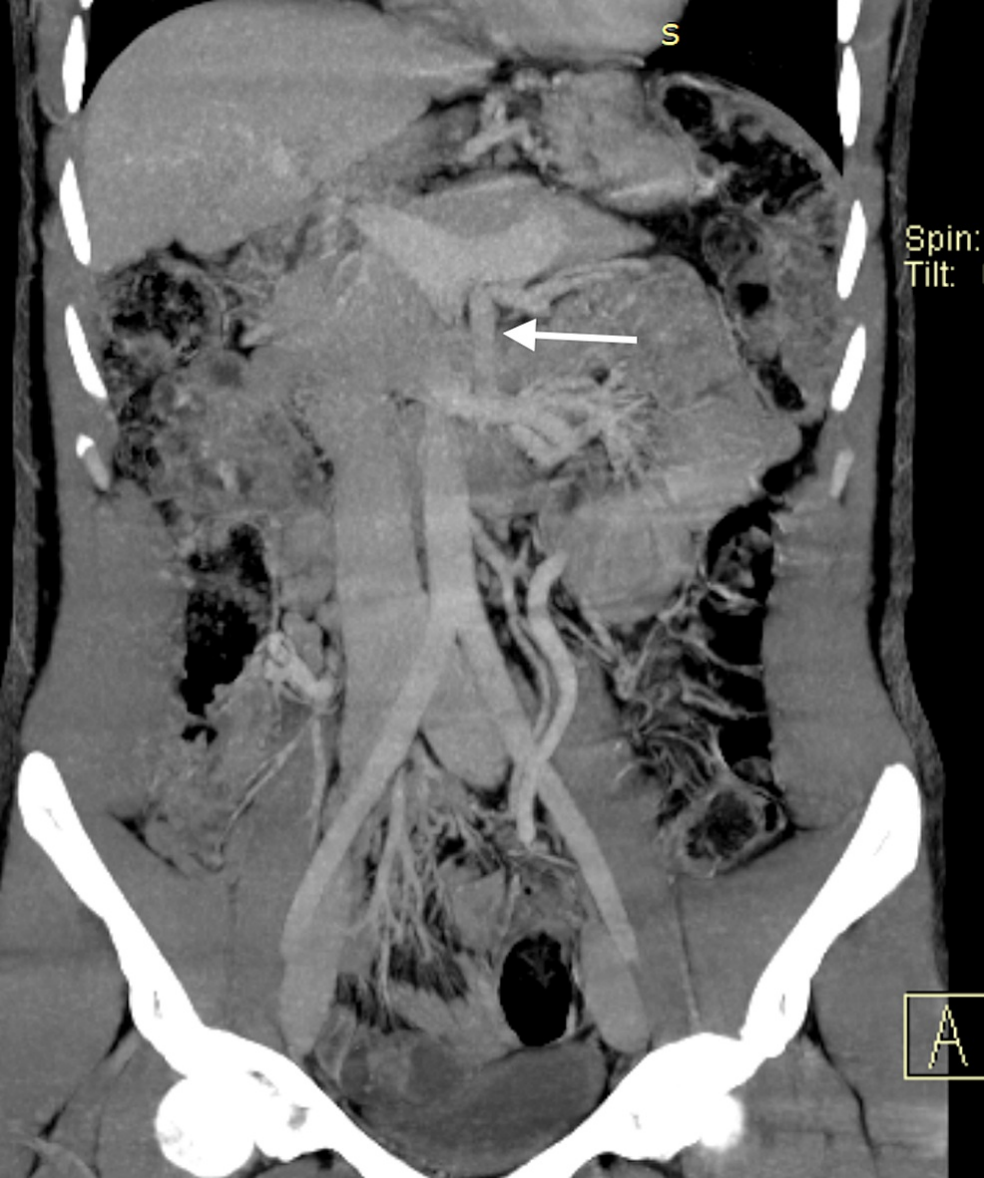
Coronal section of abdominal CT after a splenorenal shunt. Coronal CT scan showing a patent splenorenal shunt in place (arrow).

**Figure 4:**
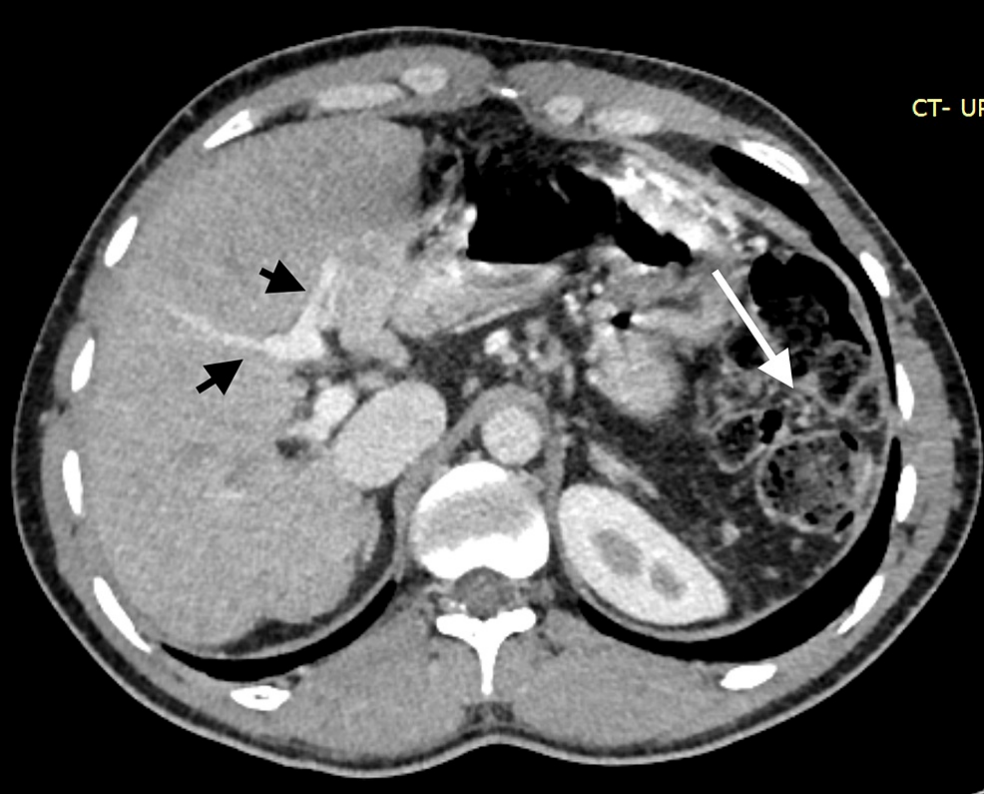
Axial abdominal CT after the shunt procedure. Axial CT scan showing normal liver parenchyma and a large left branch of the portal vein as compared to the right (arrowheads) and an absent spleen (arrow).

**Figure 5:**
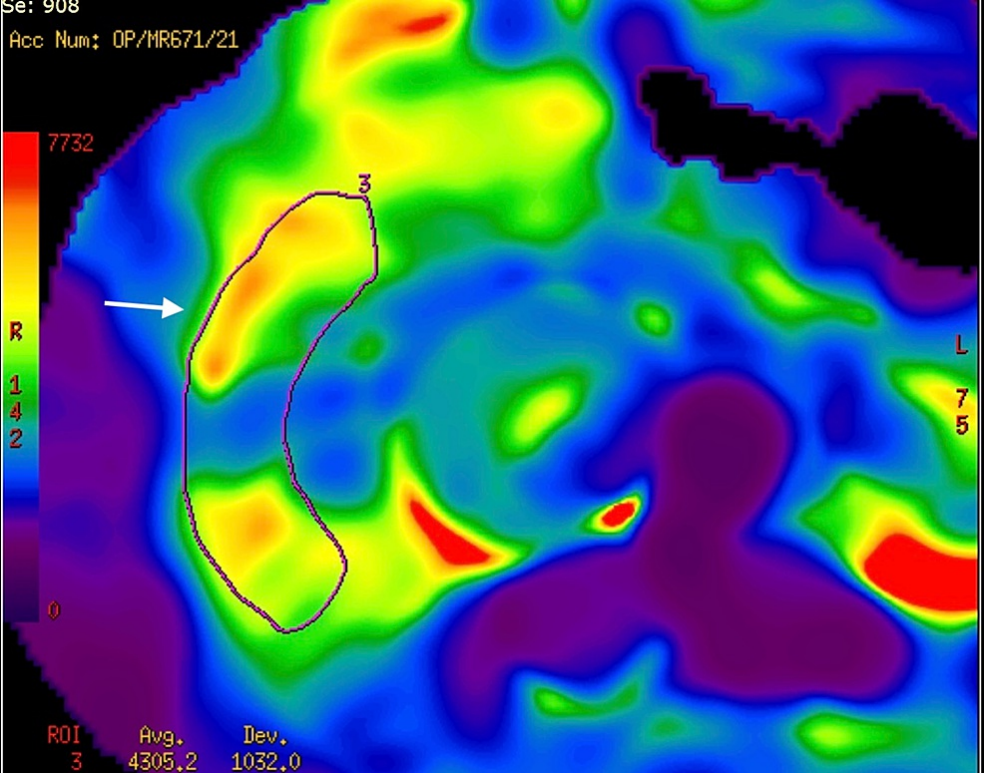
MR elastography image. MR elastography of the patient with 4.4 Kpa mean shear stiffness value of the liver, excluding cirrhosis (arrow).

A year and a half after the surgical shunt, he presented to our medical center with a complaint of trouble keeping his balance, slowness of movement, and hypophonia. Symptoms were progressive, and subsequently, he noticed that he had difficulty positioning himself in bed, keeping his balance, and walking without support. However, there was no fluctuation in symptoms. There was no reported change in mentation or bizarre behavior. There was no history of abdominal swelling, no new bleeding, or change in stool color. Family history was non-revealing for such symptoms in close relatives. His vital signs were stable on physical examination, and he had pink conjunctiva. The abdominal exam was unremarkable, and there was no edema. On neurologic assessment, he was conscious and alert. The mini-mental exam was 28/30, which is normal for his educational background. There was evident hypophonia, gait instability, and cogwheel rigidity of the lower extremities. No significant lateralization of the symptoms was noted. Gait was difficult to assess due to the marked bradykinesia during evaluation. The brain's MRI showed symmetrical hyperintensity on T1-weighted images in the globus pallidus (Figure 1). Similar symmetrical hyperintensity is also seen on fluid-attenuated inversion recovery (FLAIR) images in the crura of the midbrain bilaterally (Figure 2). CT of the abdomen showed normal liver parenchyma with patent splenic portal vein without any intraluminal filling defect (Figure 3). The portal vein's left branch was larger than the right (Figure 4). Magnetic resonance elastography showed the mean shear stiffness value of the liver to be 4.4 Kpa (Figure 5), excluding cirrhosis (cut-off value >4.65 Kpa). Liver enzymes and complete blood count (CBC) were normal. Abdominal ultrasonography with color Doppler demonstrated a regular liver echo pattern, size (14.3 cm), and smooth contour. The portal vein had a normal caliber (0.8 cm). Hepatic veins and the intrahepatic portion of the inferior vena cava (IVC) had typical diameters and flow.

With the clinical presentation and MRI findings considered, a diagnosis of AHD was made. He was put on levodopa and carbidopa 100/25 mg three times per day with dose escalation for one year. Along with a dietary adjustment, he was also started on ammonia-decreasing therapy (lactulose, L-Ornithine, L-Aspartate granules). He also continued treatment with warfarin. However, there was no significant neurologic improvement noted in this patient.

## Discussion

Though described clinically in the early 20th century, AHD as a clinical entity was first described by Victor in 1965 [3]. It is typically encountered in two clinical scenarios. The first group is patients with severe liver disease, typically advanced cirrhosis. In contrast, the second group is patients with surgical or spontaneous portosystemic shunts. The condition has not been well described in the Sub-Saharan Africa context outside South Africa, a part of the world with a high prevalence of schistosomiasis and associated portal hypertension [4].

The pathogenesis of AHD remains unclear, but evidence suggests the condition is related to metal deposition. In particular, brain manganese (Mn) overload may contribute to the etiopathogenesis of the condition. The liver and biliary system clear manganese from both blood and CSF. In patients with AHD, manganese levels were elevated compared to healthy controls [5]. With shunt or significant liver dysfunction, manganese deposition in the subcortical parts of the brain induces neuronal loss, leading to predominant extrapyramidal symptoms [6].

The clinical picture of AHD usually involves an insidious, chronic progressive course, although an acute presentation has also been described [7]. Typically, patients could present with extrapyramidal symptoms, neuropsychiatric cognitive impairment, and myelopathy. Symptoms include ataxia, tremor, chorea, dysarthria, parkinsonism, dystonia, and myoclonus [8]. The general choreoathetotic symptoms might mimic Huntington's disease in some patients, and a misdiagnosis can be made. Our patient's clinical features of AHD started after 18 months of splenorenal shunt procedure, which aligns with similar reports in the literature. As our patient also lacks features of liver cirrhosis and decompensation, Wilson's disease or hepatic encephalopathy are unlikely, though both need to be considered in such scenarios.

The diagnostic modalities used to confirm or exclude AHD diagnosis involve a combination of clinical examination and neuroimaging [9]. In chronic AHD specifically, MRI often shows basal ganglia changes with hyperintense signal changes on T1-weighted imaging, usually in the globus pallidus [2]. The T1-imaging finding is associated with preferential deposition of manganese in the pallidum [5]. Blood tests can be less helpful in diagnosing chronic AHD; blood manganese levels might be variable and rarely used as a diagnostic tool. Levels were significantly higher in patients with prior portocaval anastomosis or transjugular intrahepatic portosystemic shunt (TIPS), suggesting shunting of manganese as the likely etiology [6]. In our patient, AHD resulted from the splenorenal shunt bypassing the detoxication effect of the liver, even though his liver function tests were in the normal range.

Unfortunately, treatment options for chronic AHD are limited, and in most patients, the condition is irreversible [10]. Trial of dopamine agonists for the parkinsonian symptoms may be initiated in some groups of patients; however, a significant proportion of patients do not respond to this, as was the case in our patient [11]. There have been reports of improvement in symptoms with rifaximin, a drug commonly used to treat hepatic encephalopathy; however, these studies are limited to a few case series [12]. Liver transplantation has been effective for some patients with AHD in a select group of patients [13,14]. However, since many patients present with advanced neurological symptoms, they are often excluded from the transplant waiting list.

Distal splenorenal shunt and devascularization is a standard procedure employed in managing recurrent variceal bleeding in patients with hepatosplenic schistosomiasis [15]. A well-recognized complication is a hepatic encephalopathy; however, the risk of AHD has also been described previously [16]. In sub-Saharan African countries, schistosomiasis is an endemic condition, and many patients in the continent develop portal hypertension and variceal bleeding due to chronic infection [17]. Furthermore, with the expansion of surgical services in the continent, AHD needs to be recognized as a possible complication of such shunt procedures.

## Conclusions

AHD is a rare complication in advanced cirrhotic patients or patients with any form of a portosystemic shunt. Brain manganese overload plays a significant role in developing the condition, leading to progressive neurologic impairment not easily treatable with available modalities. Surgical management of schistosomiasis might be complicated with such neurologic problems, and careful consideration before a procedure is of paramount importance. If AHD develops, recognizing the condition helps plan therapy goals in patients with advanced liver disease or portosystemic shunt.
